# Harmful Elements (Al, Cd, Cr, Ni, and Pb) in Wild Berries and Fruits Collected in Croatia

**DOI:** 10.3390/toxics6020031

**Published:** 2018-06-08

**Authors:** Michaela Zeiner, Iva Juranović Cindrić

**Affiliations:** 1 Man-Environment-Technology Research Centre, School of Science and Technology, Örebro University, Gymnastikgatan 1, 70182 Örebro, Sweden; 2 Division of Analytical Chemistry, Department of Chemistry, BOKU—University of Natural Resources and Life Sciences, Muthgasse 18, 1190 Vienna, Austria; 3 Department of Chemistry, Faculty of Science, University of Zagreb, Horvatovac 102a, 10000 Zagreb, Croatia; ijuranovic@chem.pmf.hr

**Keywords:** blueberries, lingonberries, rose hips, aluminium, cadmium, chromium, nickel, lead, provisional tolerable intake

## Abstract

Fruits and vegetables are considered a beneficial contribution to the human diet. Especially, berries contain a great deal of bioactive compounds, such as anthocyanins, organic acids, tannins, phenols, and antioxidants. Apart from organic substances, inorganic nutrients are also present in fruits. Some metals and metalloids are essential for humans, whilst others may exhibit harmful effects. Wild grown berries, collected in so-called unpolluted areas, are considered to be free of any potentially toxic ingredients. However, due to transmission processes pollutants can also reach remote areas and, furthermore, metal uptake from the soil via roots has to be taken into account. Thus, the presented study focused on the determination of Al, Cd, Cr, Ni, and Pb in lingonberries, blueberries, and rose hips collected in a non-polluted area in Croatia. Neither Cd nor Cr could be found in any sample. Ni levels were mainly up to 25 mg/kg, in a comparable range to the literature data. No health threat is to be expected by eating these fruits and berries regarding Cd, Cr, and Ni. Rose hips, however, contain Pb beyond the stipulated limit in fruits, and also Al is present at a high level (8 mg/g).

## 1. Introduction

Berries have been collected for nutritional purposes for a long time. They can be either consumed directly as fresh or dried fruits or in various processed forms, such as jams, syrups, infusions, juices, and jellies, or as ingredient of yoghurts or ice-creams. Diet in the Western world includes vegetables and fruits, as well as other plant parts, such as roots, leaves, stems, and seeds, from more than 40 botanical families [1]. Many demonstrate a presence of phytochemicals in fruit and vegetables, which contribute to good human health by influencing metabolic processes [1], e.g., free radical scavenging, stimulating the immune system, inducing apoptosis, and detoxification. Including fruits and vegetables in the daily diet is recommended in dietary guidelines worldwide. However, the consumers’ awareness of nutraceutical components is increasing. Thus, ongoing scientific interest in berries, especially wild grown ones, is needed [2]. Regarding public interest, it was estimated in the 1990s, that one family gathers approx. 20–30 kg of berries per year [3]. Even if gathering berries and fruits by oneself is nowadays still carried out, it has started to change from self-collecting in the woods to the usage of pick-your-own farms, which offer a wide range of berries, such as strawberries, raspberries, or blackberries, and processed products [4,5].

Berry fruits, in general, contain a wide range of micro- and macronutrients, such as fibres, minerals, vitamins, and folate, but their biological properties have been attributed mainly to hydrophilic phenolic-type phytochemicals. Usually not only one bioactive compound determines the positive effects of a fruit, but the presence of multiple phytochemicals exhibiting complementary, synergistic, and/or additive effects results in the wide spectrum of health promoting effects [1,2]. Thus, berry research has been focused mainly on organic composition [6,7,8,9,10,11].

Regarding inorganic analytes, investigations dealing with nutrients in different berry types, e.g., major and minor essential elements, have been published for decades [12,13,14,15,16,17]. Using old data, such as from 1944 [12] or 1974 [13], it has to be taken into account that the analytical methodology, as well as the environmental influences, have changed, reducing their significance for current evaluations. In addition to the nutritional value, the content of minerals present also influences food quality parameters, like appearance, taste, texture, stability, and even flavonoid production [18]. Conversely, ripeness affects the mineral composition, and some nutrients have been found to decrease with maturity [15], whereas the level of the harmful element arsenic increased [16]. Furthermore potentially harmful metals and metalloids have to be taken into account. Berries collected in highly contaminated areas have been analysed for this purpose [3,19,20]. Additionally, in remote areas heavy metal pollution may occur due to transmission processes. Thus, food safety is of concern [21]. Ten chemicals have been classified to be of major public health concern, including four metals/metalloids (arsenic, cadmium, lead, and mercury) [22]. In addition to the top-ten, other metals may exhibit harmful effects to humans through food intake, e.g., nickel [23,24]. Therefore, maximum allowed limits have been established for harmful elements, and food on the market should not contain certain metals in levels that might result in exceeding the respective allowable intakes. PTWI (provisional tolerable weekly intake), PTDI (provisional tolerable daily intake), or PTMI (provisional tolerable monthly intake) levels have been published by the FAO/WHO [25].

The aim of the present investigation was to determine the content of five elements: aluminium (Al), cadmium (Cd), chromium (Cr), nickel (Ni), and lead (Pb) in wild berries and fruits, since they are considered to be potentially toxic contaminants. Regarding the methodology, previously optimised digestion and measurement procedures were applied, i.e., acidic microwave-assisted digestion followed by inductively-coupled plasma atomic emission spectrometry (ICP-AES) [26]. Closed-vessel microwave digestion as a sample preparation method has been approved for the determination of 31 elements in foodstuffs [27].

## 2. Materials and Methods

### 2.1. Chemicals and Glass/Plastic Ware

Reagents for sample and CRM digestion, i.e., hydrogen peroxide (H_2_O_2_) and supra pure nitric acid (HNO_3_) were purchased from Sigma (Munich, Germany). The standard stock solution used for preparation of calibration standards (ICP Multielement Standard IV) was obtained from Merck (Darmstadt, Germany). Ultra-pure water, resistivity ≥ 18 MΩ·cm, was prepared by in-house equipment. Quality control measurements were based on the certified reference material strawberry leaves (CRM: LGC7162) from LGC Standards (Middlesex, UK). All glass- and plasticware used for sample storage and preparation were pre-cleaned with diluted nitric acid.

### 2.2. Samples and Sample Preparation

Wild berries and fruits, namely blueberries (*Vaccinium myrtillus*), lingonberries (*Vaccinium vitis-idaea*), and rosehip (*Rosa canina*), were collected during summer and fall 2010 when optimally ripe in a non-polluted rural area of Croatia (Slunj: N, 45.07°; E, 15.36°; A: ~280 m). The sampling site covered approx. 200 m^2^. For each fruit type five plants, randomly distributed in the sampling area, were sampled by collecting 5 to 15 fruits. Immediately after picking, the fruits were frozen and kept at −20 °C prior to further work-up. After thawing the fruits were rinsed with ultra-pure water. Rose hips were separated into flesh (mesocarp) and seeds. All fruits were dried at 105 °C for 24 h. Using a metal-free mortar, the samples were then ground and homogenised, resulting in a pooled sample for each plant (i.e., five subsamples for each fruit type) In order to destroy the organic matrix, all samples underwent a previously-optimised acidic microwave assisted digestion [26], whereby approx. 0.25 g to 0.5 g (weighed to the nearest 0.1 mg) were treated with 5 mL nitric acid (*c* = 7 mol/L), applying the following three-step digestion procedure: (1) 150 °C/10 min, (2) 160 °C/10 min, and (3) 190 °C/20 min. The obtained clear solutions were then brought to 10.0 mL with ultrapure water.

### 2.3. Apparatus and Measurements

Microwave-assisted digestion of the fruits, CRM, and blanks were done in a MWS-2 Microwave System Speedwave instrument (Berghof Laborprodukte GmbH, Eningen, Germany). The subsequent quantitative determination of metals and metalloids was carried out using a Prodigy High Dispersive ICP-AES spectrometer (Teledyne Leeman, Hudson, NH, USA), using a simultaneous mode, the optimal instrumental conditions are listed in Table 1. The emission lines selected along with the respective characteristics of the analytical method are given in Table 2.

External standards in the concentration range from 0.0500 mg/L to 5.00 mg/L were used to calibrate all analytes. The standard solutions were prepared by diluting a multi-element standard stock solution (1000 mg/L) with 1% *w*/*w* HNO_3_. This diluted nitric acid is also the medium of the blank solution, whose intensity was subtracted from all sample values for blank correction. In case of sample concentrations outside the calibration range, appropriate dilutions to the sample were performed with 1% *w*/*w* HNO_3_.

### 2.4. Calculations

All solutions, i.e., fruit and berry digests, blanks, and CRM digest were measured thrice. The blank-corrected values (mass concentrations) were converted into contents in mg/kg dried plant material considering dilution factor, final volume, and mass of dry matter. Finally, means and standard deviations were calculated for all samples. In order to see statistically significant differences between the three fruits analysed, ANOVA test was carried out, based on *p* < 0.05. All calculations were performed using Microsoft Excel 2010 and 2013.

### 2.5. Characterisation of the Analytical Method

In order validate the analysis, parameters of trueness, precision, and day-to-day repeatability were determined from strawberry leaves which were chosen as a certified reference material for plant matrix. The samples were analysed after calibration on two different days. The recoveries were obtained using the following formula:

$${\mathrm{recovery}}_{x}\mathrm{in}\;\%=\;\frac{{\mathrm{content}}_{x,\;\mathrm{found}}\;\mathrm{in}\;\mathrm{mg}/\mathrm{kg}}{{\mathrm{content}}_{x,\;\mathrm{certified}}\;\mathrm{in}\;\mathrm{mg}/\mathrm{kg}}\times 100$$

Based on Bouman’s procedure [28], the limits of detection (LOD; 3σ) and quantification (LOQ; 10σ) were calculated.

## 3. Results

### 3.1. Analytical Method

The limits of detection (LOD) for the dried fruits, based on a digested mass of 0.25 g and a final volume of 10.0 mL, are below 0.3 mg/kg for all analytes except for Al, whose LOD is 2.9 mg/kg. In addition to the LOD values, the recoveries obtained by analysing the CRM (ranging from 85% to 113%) are given in Table 2. These figures of merit are in the range of reported ones for similar studies [26,29]. The coefficients of determination (*R*^2^) of the calibration curves are all beyond 0.9995. The precision presented as relative standard deviation (RSD) for triplicate measurements is below 1.2% for all analytes. The RSD obtained for the day-to-day-repeatability is not higher than 2.5%.

### 3.2. Elemental Content of Wild Berries and Fruits

The results obtained for the three kinds of berries and fruits for the five metals analysed are summarised in Table 3. In all samples the Cd, as well as Cr, levels were below the respective LODs. For the other analytes (Al, Ni, Pb) the minimum, mean, and maximum values are given. Statistically significant differences in the element content were found for Al and Ni, the *p*-values are 2.6 × 10^−14^ and 0.0023, respectively. Conversely, the Pb contents do not differ statistically significantly between the three sample types analysed (*p* = 0.24)

## 4. Discussion

### 4.1. Analytical Method

The above described figures of merit for the analytical method applied are in the range for the determination of trace elements in biological samples. LODs in a similar range were also obtained by other working groups [17,29]. Limit values for Cd and Pb in fruits and vegetables are stipulated (see Table 4). For both metals the calculated LOD is lower than the maximum allowed content. The latter are given for fresh weight. Since the water content of fruits and berries is around 90% [14], the elemental content referred to fresh weight is 10 times lower than referred to dry weight. Based on the characteristic data found, the analytical method chosen has been proven to be appropriate for the given analytical task.

### 4.2. Elemental Content of Wild Berries and Fruits

Based on the five elements determined, all three fruit types differ statistically significantly from each other regarding Al and Ni content. In the following all elements analysed are discussed in detail.

Aluminium has no known physiological role in the human body. Until the 1970s it was considered to be innocuous, but starting in the 1980s toxic effects to animals, plants, and humans have been reported [36]. Al rarely shows acute toxicity, but chronic intoxications have to be considered, especially due to the fact that Al accumulates with age [37,38]. Diet is regarded as being a significant contributor to the body burden of aluminium [39], and its weekly intake is limited by the FAO/WHO [25]. The Al content found in the fruits and berries analysed was highest in rose hips, followed by blueberries and, finally, lingonberries, the mean values being 8242 mg/kg, 1248 mg/kg, and 42.5 mg/kg, respectively. The data differ from each other in a wide range, even since all samples were collected in the same area having a similar environmental background. Thus, it can be clearly seen that the uptake differs between the plants. Nile and Park do not give any value for Al in berries in their review on berry composition, they just mention that berries are rich in Al [10]. Rose hips used for the preparation of infusions collected in Turkey show a mean Al content of 157 mg/kg [40], whereby it is not reported if the entire fruit or only the flesh (as in the present study) was analysed. However, comparable Al contents were found in strawberries from Pakistan (Lahore region), whereby the values decreased during ripeness from 740 mg/kg to 230 mg/kg d.w. [15]. The PTWI given by the FAO/WHO limits the weekly Al dietary intake with 1 mg/kg bw. Thus, an 80 kg-person should not consume more than 100 g of rose hips per week. For blueberries and lingonberries more than 600 g, and even 2 kg, are to be eaten resp. in order to reach the maximum, considering no other Al sources. Chinese blueberries contained 41 mg/kg [41], much lower than the results from this study, but in the same order of magnitude than the data obtained for lingonberries. This fact can be explained by the impact of bedrock composition on Al content in plants. A potential health threat might only be caused by rose hip intake. This is a drawback, since rose hips have recently been defined as a functional food based on bioactive ingredients, as well as due to their content of essential elements, such as Ca, Mg, K, S, Si, Se, Mn, and Fe [42].

Cadmium, being a highly toxic metal, is one of ten chemicals classified by the WHO to be a major public health concern [22]. It occurs naturally in soil, but it is also a pollutant in the environment due to anthropogenic impact. Since it is easily taken up and accumulated by plants and crops through the root systems, it may easily enter food [43], especially berries and fruits. In all samples, including blueberries, lingonberries, as well as rose-hips, no Cd was detected, meaning that the contents are below the LOD of 0.028 mg/kg d.w. Commission Regulation (EU) No. 2015/1005 limits Cd in fruits and vegetables to 0.050 mg/kg f.w., corresponding to 0.50 mg/kg d.w. [30]. Thus, all fruits analysed are supposed to be safe for humans regarding this element. The PTMI is 0.025 mg/kg bw, which would be 2 mg per month for an 80 kg person. Considering Cd present in the berries at the LOD level, this corresponds to more than 700 kg of fresh blueberries in one month. An investigation of blackberries, elderberries, autumn olives, and candleberry-myrtles revealed that they also contained Cd only below LOD [17]. Whereas no Cd was found in Azorean blueberry, levels between LOD and limit of quantitation (LOQ) were registered for Madeiran blueberry [17]. A Russian study reports Cd in blue- and cranberries in the range from 0.03 mg/kg to 0.06 mg/kg d.w. [3]. Strawberries collected in Serbia did not contain Cd at detectable concentrations either [29]. Blueberries collected in China were reported to contain 0.034 mg/kg Cd [41], slightly above the LOD of the current study. Rose hips from Turkey have been reported to contain quite a high amount of Cd, namely 0.81 mg/kg d.w. [44]. A working group from Bangladesh found Cd contents ranging from 0.012 mg/kg and 0.216 mg/kg in leafy and non-leafy vegetables [43].

Chromium, as Cr(III), plays a crucial role in human health and is, thus, defined as an essential trace element [45]. Special attention has to be drawn to its interaction in glucose tolerance and Cr supplementation in persons with diabetes, hypoglycaemia, and obesity [46]. In addition to the positive effects of Cr(III), Cr(VI) is a toxic, carcinogenic substance, this fact being reflected in the 500-times lower reference oral dose for the latter species [32,33] compared to the former species. No limit for Cr in foods is given in the Codex Alimentarius Commission, or by Australia, New Zealand, Japan, the United States, and Taiwan [41]. Even if Cr(III) is reported to be mainly found in fruits, vegetables, and grain products [47,48], no Cr could be detected in all samples of this investigation (<0.018 mg/kg). Data for Cr in blueberries are given by Hua et al., who found 0.77 mg/kg [41]. Additionally, in fresh strawberries the Cr content is quite low, ranging from 0.01 mg/kg up to 0.03 mg/kg [29]. Wild berries from Portugal, analysed by Llorent-Martínez and colleagues, found Cr in blackberries, elderberries, autumn olives, and candleberry-myrtles up to 0.6 mg/kg fresh weight [17]. Cranberries from Russia have been reported to contain Cr from 0.02 mg/kg up to 1.5 mg/kg, and blueberries 0.03 to 0.06 mg/kg d.w. [3]. Literature data for rose hips are given by Duran and colleagues, who found 0.80 mg/kg d.w. Cr [44].

Nickel is naturally occurring in soils ranging from 4 to 80 mg/kg in the USA [49] and, for agricultural soil in Velika Gorica, Croatia (N 45.7173°–E 16.0571°; A 107 m), a mean content of 55 mg/kg was found [50]. The main exposure route to nickel is via food intake, approx. 0.100 to 0.300 mg/day are taken up by per adult [23,49]. Apart from contact dermatitis, genotoxicity, haematotoxicity, teratogenicity, immunotoxicity, and carcinogenicity have been identified as harmful effects of Ni [24]. Thus, its intake is limited to 0.020 mg/kg bw per day [23,34]. In the present study the highest Ni content was found in blueberries (25 mg/kg), followed by rose hips (11 mg/kg) and lingonberries (2.5 mg/kg). The daily consumption of 100 g fresh blueberries would lead to an intake of 0.250 mg, being approx. 16% of the allowed limit for an 80 kg person. Thus, no harmful effects are to be expected by the berries analysed. Cranberries from polluted areas (close to a Ni-Cu-smelter) showed elevated levels (up to 97 mg/kg d.w.), whereas the background contents were in the same range as the results from the current study, i.e., 2 mg/kg to 9 mg/kg [3]. No high Ni accumulation in lingonberries collected in the vicinity of a chromium mine was reported by a Finnish research group [51]. Blueberries from the same contaminated area did not accumulate Ni that much, with contents being in the range from 4 mg/kg to 11 mg/kg for all sampling sites [3]. A Chinese working group found 2.2 mg/kg d.w. Ni in blueberries [41]. Wild berries from Portugal contain Ni in the same order of magnitude, namely from 0.6 mg/kg to 1.6 mg/kg [17], as our results for lingonberries. Additionally, results for strawberries are in the same range [29]. Regarding rose hips, the literature data are available for Turkish fruits, with the mean content being 6.5 mg/kg d.w. [44].

Lead is well known as a toxic element, exhibiting developmental neurotoxicity in young children and cardiovascular effects and nephrotoxicity in adults [35]. Thus, the Pb content in fruits and berries is limited to 0.10 mg/kg f.w. and 0.20 mg/kg f.w., respectively [35], whereas the PTI value was withdrawn in 2010 [22]. The highest maximum and mean level for Pb were found in rose hips (15 mg/kg; 3.3 mg/kg, resp.), exceeding the limit value in foods, which would correspond to approx. 1 mg/kg d.w. This result is comparable to that form Turkish rose hips with 10 mg/kg d.w. [44]. Blueberries’ Pb content showed the smallest range, compared to rose hips and lingonberries. Only one sample exceeds the limit in berries (2.4 mg/kg > 2 mg/kg). The mean value of Pb in blueberries (1.66 mg/kg) is similar to the data from Barcan et al., who reported contents ranging from 0.7 mg/kg up to 1.5 mg/kg [3]. Conversely, the Chinese working group found less, their average being 0.135 mg/kg d.w. [41]. Lingonberries contained the least Pb of all fruits analysed, except for one sample (9.3 mg/kg) all results are below 1 mg/kg, thus being within the allowed range for berries. Even less Pb was found in lingonberries from Finland, whose contents ranged from 0.0006 mg/kg to 0.0011 mg/kg f.w. [51], corresponding to approx. 0.006 mg/kg to 0.011 mg/kg dried berries. Wild berries gathered in Portugal all had Pb levels below the LOD (0.012 mg/kg f.w.) [17].

## 5. Conclusions

Wild berries and fruits collected in a remote and, thus, considered unpolluted area in Croatia have been analysed for potentially toxic elements, revealing that no health threat is to be expected by eating these fruits and berries regarding Cd, Cr, and Ni. Due to high Al and Pb contents in rose hips, their intake should not exceed 100 g per week for an adult, especially in the case of chronic renal failure. Only in cases of high consumption of blueberries are harmful effects by Al and Pb to be expected. Lingonberries were found to have the lowest contents of all metals investigated.

## Figures and Tables

**Table 1:** Operating conditions of the Prodigy High Dispersive ICP-AES.

Parameter	Settings
Spectrometer	High resolution Echelle polychromatorLarge format programmable array detector (L-PAD)
RF-Generator	40 MHz “free-running”
Output power	1.1 kW
Argon flows	Coolant: 18 L min^−1^ Auxiliary: 0.8 L min^−1^ Nebuliser: 1.0 L min^−1^
Peristaltic pump	1.0 mL min^−1^
Nebuliser	Pneumatic (glass concentric)
Spray chamber	Glass cyclonic
Plasma viewing	Axial
Sample uptake delay	30 s

**Table 2:** Characteristics of the analytical method.

Analyte	Wavelength (nm)	LOD in Digest Solution (mg/L)	LOD in Dried Fruits (mg/kg)	Recovery (%)
Aluminum	308.215	0.074	2.9	110
Cadmium	214.441	0.00071	0.028	113
Chromium	206.149	0.00045	0.018	101
Nickel	231.604	0.0038	0.15	85
Lead	220.353	0.0070	0.28	101

**Table 3:** Minimum–mean–maximum metal content in fruit material (mg/kg d.w. ^1^), n = 5.

Metal	Lingonberries	Rose Hip	Blueberries
Aluminum	34.9–42.5–63.9	7527–8242–8836	1093–1248–1463
Cadmium	<0.028	<0.028	<0.028
Chromium	<0.018	<0.018	<0.018
Nickel	1.81–2.49–12.9	10.6–11.3–23.5	21.0–24.8–56.2
Lead	0.542–0.601–9.28	3.00–3.34–15.3	1.19–1.66–2.42

^1^ d.w. = dry weight.

**Table 4:** Limits for metals in food.

Metal		Limit in Food		Intake Limit ^1^	Lit.
Aluminum				1.00 mg/kg bw/week (PTWI)	[25]
Cadmium		Fruits and vegetables	0.050 mg/kg f.w.		[30]
				0.025 mg/kg bw/month (PTMI)	[22]
				0.001 mg/kg bw/day (Rfd)	[31]
Chromium	insoluble Cr(III)-salts			1.500 mg/kg bw/day (Rfd)	[32]
	Cr(VI)			0.003 mg/kg bw/day (Rfd)	[33]
Nickel	soluble salts			0.020 mg/kg bw/day (Rfd)	[34]
				0.020 mg/kg bw/day (Trv)	[23]
Lead		Fruit, excluding cranberries, currants, elderberries and strawberry tree fruit	0.10 mg/kg f.w.		[35]
		cranberries, currants, elderberries and strawberry tree fruit	0.20 mg/kg f.w.		[35]
				PTI value withdrawn 2010	[22]

^1^ f.w. = fresh weight; bw = body weight; PTWI = Provisional tolerable weekly intake; PTMI = Provisional tolerable monthly intake; Rfd = Reference oral dose; Trv = Toxicity reference value.
